# The effects of asking a fertility intention question in primary care settings: a systematic review protocol

**DOI:** 10.1186/s13643-017-0412-z

**Published:** 2017-01-19

**Authors:** Paul A. Henning, Carolyne K. Burgess, Heidi E. Jones, Wendy V. Norman

**Affiliations:** 1 Department of Obstetrics and Gynaecology, University of Calgary, Calgary, Canada; 2 School of Population and Public Health, |University of British Columbia, Vancouver, Canada; 3 School of Public Health & Hunter College, City University of New York (CUNY), NY, USA

**Keywords:** Planned pregnancy, Contraception, Family planning, Pregnancy outcomes, Unplanned pregnancy, Pregnancy complications, Preconception care, Prenatal care

## Abstract

**Background:**

Planning for pregnancy has been associated with reduced unwanted pregnancies and improved pregnancy outcomes. Despite the benefits of planned pregnancy, there are no guideline recommendations on routine counseling regarding pregnancy intention in primary care settings. The objective of the systematic review is to determine the effectiveness of incorporating questions of pregnancy intention into primary care.

**Methods:**

A systematic search of the literature will be conducted for any studies comparing questions of pregnancy intention in primary care settings with no intervention or a control intervention. Types of studies will include randomized controlled trials, non-randomized trials, and observation studies. Participants will include patients of reproductive age presenting to primary health care settings. Interventions will include any assessment of fertility intention and follow-up care compared with a control group or no intervention. Outcomes will include quantitative data with rates for contraceptive uptake, and any pregnancy related outcome. Databases (Ovid MEDLINE; Pubmed; CINAHL; EMBASE; CDR/DARE databases; Web of Science; ISRCTN registry; Clinicaltrials.gov; Cochrane Library) will be searched from the year 2000 to current. Screening of identified articles and data extraction will be conducted in duplicate by two independent reviewers. Methodological quality will be assessed using the Jadad scale. Methodological quality of observational and non-randomized trials will be assessed using the Newcastle-Ottawa scale. Discrepancies will be resolved by consensus or by consulting a third author. Meta-analyses will be performed if appropriate.

**Discussion:**

Determining the effect of including questions of pregnancy intention into primary care can provide evidence for the development of clinical practice guidelines and inform primary care providers if this simple and low-cost intervention should be routinely employed. This review will also identify any gaps in the current literature on this topic and provide direction for future research in this area of study.

**Systematic Review Registration**: PROSPERO CRD42015019726

**Electronic supplementary material:**

The online version of this article (doi:10.1186/s13643-017-0412-z) contains supplementary material, which is available to authorized users.

## Background

Sexual and reproductive healthcare are important components of overall health and should be addressed on a regular basis in the context of ongoing medical care. Taking steps to achieve one’s own fertility intentions is a vital part of reproductive health and is associated with improved pregnancy outcomes. In contrast, unintended pregnancies can have serious health, economic, and social consequences for women and their families [1]. In the United States half of pregnancies (51%) are unintended and 42% of this end in abortion [2, 3]. Similarly high rates of unintended pregnancy are found in developing countries with up to 64% of pregnancies being unintended in South America [4]. Unintended pregnancy has been identified as a human rights concern. At the 1994 International Conference on Population and Development (ICPD) held in Cairo, the Programme of Action stated that “[a]ll couples and individuals have the basic right to decide freely and responsibly the number and spacing of their children and to have the information, education and means to do so” [5].

A variety of improved outcomes have been associated with planned pregnancy. Planned pregnancy has been associated with reduced maternal risk behaviors, increased antenatal care, improved birth outcomes, decreased infant and child mortality, increased breastfeeding, decreased maternal mortality, and decreased unsafe abortion [6]. Studies have also linked wanted pregnancy to reduced long-term social and health impacts on older children, adolescents, and adults [7, 8].

Despite the benefits of planned pregnancy, there are no guideline recommendations on the provision of routine counselling regarding pregnancy intention in primary care settings. In 1995, the U.S. Preventive Services Task Force (USPSTF) included counseling as a recommended intervention to prevent unintended pregnancy. However, this recommendation was absent from subsequent published recommendations [9].

The objectives are to (1) systematically identify and assess studies on the effectiveness of routinely asking individuals of reproductive-age in primary care settings about their fertility plans; (2) synthesize evidence on incorporating a question on pregnancy intention into primary care; (3) provide evidence to primary care organizations to inform the development of practice guidelines; and (4) provide a framework for intervention strategies to support individuals to realize their fertility intentions.

## Methods and design

The review protocol was registered with the PROSPERO International Prospective Register of Systematic Reviews (Registration Number CRD42015019726, 31/07/2015). The review protocol has been approved by the Children’s and Women’s Hospital, University of British Columbia, Research Ethics Review Board (H15-01404).

The following criteria were applied when considering studies for this review:

- Types of studies
- The review will consider studies evaluating the effectiveness or efficacy of the incorporation of patient’s fertility intentions into their primary care. The studies must compare the intervention with no intervention or with a control intervention. Randomized trials, non-randomized trials, and observational studies will be eligible for inclusion.
- Types of participants
- Patients of reproductive age presenting to primary health care settings, defined as a health care setting that is the first point of care for undifferentiated patients with an undiagnosed condition or concern.
- Types of interventions
- Assessment of fertility intention and follow-up care. Examples include asking patients what their pregnancy or fertility intentions are for the coming year or the development of a reproductive life plan and subsequent preconception or contraception counseling, care, and/or referral as appropriate.
- Types of outcome measures
- Results must include quantitative data for outcomes measured.
- Primary outcomes
- Rates for contraceptive uptake, and any pregnancy related outcome, including unwanted pregnancy, unintended pregnancy, mistimed pregnancy, adverse pregnancy outcomes, and healthy maternal and newborn outcomes.

### Search methods for identification of studies

Extensive searches will be performed by two of the authors to collect all relevant studies. We will include both peer-reviewed journal articles and the gray literature in our searches. Only the English-language literature will be included in the searches.

- Search strategy
- Articles published since 2000 in English and indexed in the following databases will be searched: Ovid MEDLINE; Pubmed; CINAHL; EMBASE; CDR/DARE databases; Web of Science; ISRCTN registry; Clinicaltrials.gov; Cochrane Library. Additionally, the references of highly relevant articles will be hand-searched. Keywords and medical subject heading (MeSH) terms will be used. Additional file 1 shows the main search strategy that will be used for MEDLINE and will be modified for other databases.

### Data collection and analysis

#### Selection of studies

Titles and/or abstracts of studies retrieved using the search strategy and those from hand-searching citations of highly relevant articles will be screened independently by two review authors to identify studies that potentially meet the inclusion criteria outlines above. The full text of these potentially eligible studies will be retrieved and independently assessed for eligibility by two review team members. Any disagreement between them over the eligibility of particular studies will be resolved through discussion with a third reviewer.

#### Data extraction and management

References will be managed using Thomson ISI ResearchSoft EndNote X2 software (Thomson Reuters, New York, NY, USA). A pre-piloted form will be used to extract data from the included studies for assessment of study quality and evidence synthesis (see form in Additional file 2). Extracted information will include: study setting; study population and participant demographics and baseline characteristics; details of the interventions and control conditions; study methodology; recruitment and study completion rates; outcomes and times of measurement; indicators of acceptability to users; suggested mechanisms of intervention action; information for assessment of the risk of bias. Two reviewers will extract data independently, discrepancies will be identified and resolved through discussion (with a third author where necessary). Missing data will be requested from study authors.

#### Assessment of methodological quality in included studies

For randomized trials, two review authors will independently assess methodological quality in included studies by considering the following characteristics using the Jadad scale [10]:

- Was the study described as randomized?
- Was the method of randomization described and appropriate?
- Was the study described as double blind?
- Was the method of double blinding described and appropriate?
- Were withdrawals and dropouts described?

For observational and non-randomized trials, two review authors will independently assess methodological quality using the Newcastle-Ottawa scale. Included case control studies will be assessed by considering the following characteristics:

- Selection of study groups: is the case definition adequate? Are the cases representative? From where are controls selected? Are controls adequately defined?
- Comparability of groups: are cases and controls comparable on the basis of the design or analysis? Ascertainment of exposure/outcome: how is the exposure ascertained? Is the same method of ascertainment of exposure used for cases and controls? Is the non-response rate the same for cases and controls?

Included cohort studies will be assessed by considering the following characteristics:

- Selection: is the exposed cohort representative of the general population? Is the non-exposed cohort drawn from the same community as the exposed cohort? How is the exposure ascertained? Is it demonstrated that the outcome of interest was not present at the start of the study?
- Comparability: are the cohorts comparable on the basis of the design or analysis?
- Outcome: how is the outcome assessed? Was the follow-up long enough for outcomes to occur? Was the follow-up of cohorts adequate?

Disagreements between the review authors over the risk of bias in particular studies will be resolved by discussion, with involvement of a third review author where necessary. Any deviations from the protocol will be acknowledged and justification for any deviations will be made.

#### Data synthesis

We will provide a text summary of the findings from the included studies. We will present the results of the review by summarizing the approaches to fertility intention questions that were tested and comparing their effects on reproductive health outcomes. We will provide summaries of intervention effects for each study by calculating risk ratios, prevalence ratios or odds ratios (for dichotomous outcomes), or standardized mean differences (for continuous outcomes).

We anticipate that there will be limited scope for meta-analysis because of the range of different outcomes measured across the small number of existing trials. However, where studies have used the same type of intervention and comparator, with the same outcome measure, we will pool the results using a random-effects meta-analysis, with standardized mean differences for continuous outcomes and risk ratios for binary outcomes, and calculate 95% confidence intervals and two-sided *P* values for each outcome.

In studies where the effects of clustering have not been taken into account, we will adjust the standard deviations for the design effect. All results will be subject to double data entry. We will test for clinical heterogeneity by considering the variability in participant factors among trials and trial factors. Statistical heterogeneity will be assessed using the standard Chi-squared (significance level: 01). If high levels of heterogeneity exist (*P* < 0.1) the study design and characteristics of the study with be assessed. If low levels of heterogeneity exist (*P* > 0.1) then a meta-analysis as described above will be used to pool the results. Where statistical pooling is not possible, the findings will be presented in narrative form including tables and figures to aid in data presentation where appropriate. A funnel plot will be created by making a scatter plot of the estimated effects of the trials identified. Egger’s test will be used to examine funnel plot asymmetry to investigate possible publication or reporting bias [11]. Egger’s test will only be conducted if a minimum of ten studies are included within the meta-analysis.

The Preferred Reporting Items for Systematic review and Meta-Analysis Protocols (PRISMA-P) checklist was used to ensure that appropriate components of a systematic review protocol are included [12] (see Additional file 3).

## Discussion

Planning for pregnancy has been shown to improve pregnancy outcomes and reduce unintended pregnancies. Inclusion of a question of fertility intention into primary care encounters may improve the proportion of planned pregnancies and represent a simple and low-cost intervention to reduce unplanned pregnancies. Planning for pregnancy has been recognized as a significant public health issue that continues to be poorly addressed [9]. This systematic review will provide evidence for practice guidelines regarding the inclusion of questions of fertility intention in primary care settings and will provide a framework for developing and testing primary care intervention strategies to support individuals to optimally realize their fertility intentions.

## Additional files

Additional file 1: Table S1. MEDLINE search strategy, modified as needed for other databases (DOCX 482 kb).
Additional file 2: Appendix 1. Data Collection Form. Data collection form used to collect data from review of full text articles (DOCX 489 kb).
Additional file 3: PRISMA-P 2015 checklist-Henning.pdf. Completed PRISMA-P checklist (PDF 115 kb).

## Abbreviations

ICPD: International Conference on Population and Development
PRISMA-P: Preferred reporting items for systematic review and meta-analysis protocols
USPSTF: U.S. Preventive services task force
