# Supplementary material for: The effects of asking a fertility intention question in primary care settings: a systematic review protocol
Source: Syst Rev. 2017 Jan 19;6:11. doi: 10.1186/s13643-017-0412-z (PMC5248461; doi:10.1186/s13643-017-0412-z)
Supplement: Additional file 1: Table S1. — MEDLINE search strategy, modified as needed for other databases (DOCX 482 kb). [file 13643_2017_412_MOESM1_ESM.docx]

**Table 1** - Medline search strategy, modified as needed for other databases

Database: Ovid MEDLINE(R) In-Process & Other Non-Indexed Citations and Ovid MEDLINE(R) <1946 to Present>

Search Strategy:

--------------------------------------------------------------------------------

1     exp fertility/ or exp pregnancy/ (766443)

2     exp motivation/ or intention/ (134780)

3     exp Reproductive Behavior/ (7184)

4     exp Contraception/ (22838)

5     exp Pregnancy, Unplanned/ (1020)

6     2 or 3 or 4 or 5 (161358)

7     1 and 6 (14836)

8     ((pregnan* or proceat* or conceive* or fertil* or conception) adj3 (intent* or intend* or plan* or want* or unwant* or desire* or unplan* or contracept* or birth control*)).mp. [mp=title, abstract, original title, name of substance word, subject heading word, keyword heading word, protocol supplementary concept word, rare disease supplementary concept word, unique identifier] (16062)

9     7 or 8 (27237)

10     exp health surveys/ or exp health care surveys/ or exp interviews as topic/ or exp questionnaires/ (775048)

11     exp Counseling/ (34915)

12     exp Preconception Care/ (1547)

13     exp Family Planning Services/ (22953)

14     (question* or survey* or interview* or exam* or assess* or counsel* or ask*).mp. [mp=title, abstract, original title, name of substance word, subject heading word, keyword heading word, protocol supplementary concept word, rare disease supplementary concept word, unique identifier] (5126790)

15     10 or 11 or 12 or 13 or 14 (5311163)

16     9 and 15 (15754)

17     16 (15754)

18     limit 17 to yr="2000 -Current" (7869)
