# Supplementary material for: The effects of asking a fertility intention question in primary care settings: a systematic review protocol
Source: Syst Rev. 2017 Jan 19;6:11. doi: 10.1186/s13643-017-0412-z (PMC5248461; doi:10.1186/s13643-017-0412-z)
Supplement: Additional file 2: Appendix 1. — Data Collection Form. Data collection form used to collect data from review of full text articles (DOCX 489 kb). [file 13643_2017_412_MOESM2_ESM.docx]

**Appendix 1**

Data collection form

| Review title | Systematic Review of the Effects of the Incorporation of Fertility Intention Questions in Primary Care Settings |
| --- | --- |
| Study ID *(surname of first author and year first full report of study was published e.g. Smith 2001)* |  |
| Notes | |

# General Information

| Date form completed *(dd/mm/yyyy)* |  |
| --- | --- |
| Name/ID of person extracting data |  |
| Reference citation (e.g. Medline) |  |
| Study author contact details |  |
| Publication type  *(e.g. full report, abstract, letter)* |  |
| Notes: | |

# Study eligibility

| Study Characteristics | Eligibility criteria | | Eligibility criteria met? | | | Location in text or source *(pg & ¶/fig/table)* |
| --- | --- | --- | --- | --- | --- | --- |
|  |  |  | Yes | No | Unclear |  |
| Type of study | Experimental study including randomized controlled trials (RCTs) or cluster-randomized trials (CRTs). | |  |  |  |  |
|  | Quasi-experimental studies including quasi-randomized trials, controlled before-after studies (CBAs) and interrupted time series studies (ITSs). | |  |  |  |  |
|  | Observational studies including cohort, case-control and cross-sectional studies. | |  |  |  |  |
| Participants and setting | Patients of reproductive age (15-49) presenting to primary health care settings, defined as a health care setting that is the first point of care for undifferentiated patients with an undiagnosed condition or concern. | |  |  |  |  |
| Types of intervention | Assessment of pregnancy intention and follow-up care. Examples include asking women what their pregnancy or fertility intentions are for the coming year or the development of a reproductive life plan and subsequent preconception or contraception counseling and care as appropriate. | |  |  |  |  |
| Types of comparison | Patients of reproductive age in primary care settings with whom no discussion of pregnancy intention occurred. | |  |  |  |  |
| Types of outcome measures | Any pregnancy related outcome, including unwanted pregnancy, unintended pregnancy, adverse pregnancy outcomes, contraception uptake, and healthy maternal and newborn outcomes. | |  |  |  |  |
| Results | Quantitative results of the association between assessment of pregnancy intention/follow up care and pregnancy related outcomes. | |  |  |  |  |
| INCLUDE | EXCLUDE | |  |  |  |  |
| Reason for exclusion | |  | | | | |
| Notes: |  | | | | | |
|  | | | | | | |

**DO NOT PROCEED IF STUDY EXCLUDED FROM REVIEW**

# Characteristics of included studies

## Methods

|  | **Descriptions as stated in report/paper** | | **Location in text or source** *(pg & ¶/fig/table)* |
| --- | --- | --- | --- |
| **Aim of study** |  | |  |
| **Design** |  | |  |
| **Unit of observation** |  | |  |
| **Start date** |  | |  |
| **End date** |  | |  |
| **Duration of participation**  *(from recruitment to last follow-up)* |  | |  |
| **Ethical approval needed/ obtained for study** | Yes No Unclear |  |  |
| **Notes:** | | | |

## Participants and Intervention

|  | Description | | Location in text or source *(pg & ¶/fig/table)* |
| --- | --- | --- | --- |
| Population description  *(from which study participants are drawn)* |  | |  |
| Setting and context  *(including but not limited to healthcare system characteristics and health financing - e.g. user fees or financial coverage of PNC services - as well as social context, location).* |  | |  |
| Inclusion criteria |  | |  |
| Exclusion criteria |  | |  |
| Method of recruitment of participants *(e.g. phone, mail, clinic patients)* |  | |  |
| Informed consent obtained | Yes No Unclear |  |  |
| Total no. of subjects |  | |  |
| Clusters  *(if applicable, no., type, no. people per cluster)* |  | |  |
| Baseline imbalances  *(if applicable)* |  | |  |
| Withdrawals and exclusions |  | |  |
| Number of total person-years (if applicable) |  | |  |
| Missing data |  | |  |
| Outcome(s)  Definition, measure & classification | Any pregnancy related outcome, including unwanted pregnancy, unintended pregnancy, adverse pregnancy outcomes, contraception uptake, and healthy maternal and newborn outcomes. | |  |
|  |  | |  |
| Determinants | Socioeconomic | |  |
|  | Geographic | |  |
|  | Demographic (age, parity, ethnicity, marital status, immigration status) | |  |
| Confounding factors accounted for |  | |  |
| Effect modifiers accounted for |  | |  |
| Results  (specify, e.g. OR, RR, IRR)  (specify the reference group) | Crude | |  |
|  | Adjusted | |  |
| Authors’ reported limitations of study’s methods/results |  | |  |
| Scientific quality (specify tool, e.g. modified EPHPP tool) |  | |  |
| Notes: | | | |

## Other information

| **Study funding sources**  *(including role of funders)* |  |  |
| --- | --- | --- |
| **Possible conflicts of interest**  *(for study authors)* |  |  |
|  | **Description as stated in report/paper** | **Location in text or source** |
| **Key conclusions of study authors** |  |  |
| **References to other relevant studies** |  |  |
| **Correspondence required for further study information** *(from whom, what and when)* |  | |
| **Notes:** | | |
