# Supplementary material for: The effects of asking a fertility intention question in primary care settings: a systematic review protocol
Source: Syst Rev. 2017 Jan 19;6:11. doi: 10.1186/s13643-017-0412-z (PMC5248461; doi:10.1186/s13643-017-0412-z)
Supplement: Additional file 3: — PRISMA-P 2015 checklist-Henning.pdf. Completed PRISMA-P checklist (PDF 115 kb). [file 13643_2017_412_MOESM3_ESM.pdf]

PRISMA-P 2015 Checklist

This checklist has been adapted for use with protocol submissions to *Systematic Reviews* from Table 3 in Moher D et al: Preferred reporting items for systematic review and meta-analysis protocols (PRISMA-P) 2015 statement. *Systematic Reviews* 2015 **4**:1

Section/topic	#	Checklist item	Information reported		Line number(s)
			Yes	No	
**ADMINISTRATIVE INFORMATION**					
**Title**					
Identification	1a	Identify the report as a protocol of a systematic review	Yes		pg 1, line 6
Update	1b	If the protocol is for an update of a previous systematic review, identify as such		No	
**Registration**	2	If registered, provide the name of the registry (e.g., PROSPERO) and registration number in the Abstract	Yes		pg 2, line 48
**Authors**					
Contact	3a	Provide name, institutional affiliation, and e-mail address of all protocol authors; provide physical mailing address of corresponding author	Yes		pg 1, line 8-32
Contributions	3b	Describe contributions of protocol authors and identify the guarantor of the review	Yes		pg 8, line 58-60, pg 9 line 4-10
**Amendments**	4	If the protocol represents an amendment of a previously completed or published protocol, identify as such and list changes; otherwise, state plan for documenting important protocol amendments		No	
**Support**					
Sources	5a	Indicate sources of financial or other support for the review	Yes		pg 9, line 15-19
Sponsor	5b	Provide name for the review funder and/or sponsor	NA		pg 9, line 22-24

Section/topic	#	Checklist item	Information reported		Line number(s)
			Yes	No	
Role of sponsor/ funder	5c	Describe roles of funder(s), sponsor(s), and/or institution(s), if any, in developing the protocol	NA		
**INTRODUCTION**					
**Rationale**	6	Describe the rationale for the review in the context of what is already known	Yes		pg 3, line 7-49
**Objectives**	7	Provide an explicit statement of the question(s) the review will address with reference to participants, interventions, comparators, and outcomes (PICO)	Yes		pg 3, line 51-60
**METHODS**					
**Eligibility criteria**	8	Specify the study characteristics (e.g., PICO, study design, setting, time frame) and report characteristics (e.g., years considered, language, publication status) to be used as criteria for eligibility for the review	Yes		pg 4, line 20-57, pg 5, line 4-8
**Information sources**	9	Describe all intended information sources (e.g., electronic databases, contact with study authors, trial registers, or other grey literature sources) with planned dates of coverage	Yes		pg 5, line 11-33
**Search strategy**	10	Present draft of search strategy to be used for at least one electronic database, including planned limits, such that it could be repeated	Yes		Additional File 1
STUDY RECORDS					
Data management	11a	Describe the mechanism(s) that will be used to manage records and data throughout the review	Yes		pg 6, line 4-20
Selection process	11b	State the process that will be used for selecting studies (e.g., two independent reviewers) through each phase of the review (i.e., screening, eligibility, and inclusion in meta-analysis)	Yes		pg 6, line 4-20
Data collection process	11c	Describe planned method of extracting data from reports (e.g., piloting forms, done independently, in duplicate), any processes for obtaining and confirming data from investigators	Yes		pg 6, line 4-20, Additional File 2

Section/topic	#	Checklist item	Information reported		Line number(s)
			Yes	No	
**Data items**	12	List and define all variables for which data will be sought (e.g., PICO items, funding sources), any pre-planned data assumptions and simplifications	Yes		pg 6, line 4-20
**Outcomes and prioritization**	13	List and define all outcomes for which data will be sought, including prioritization of main and additional outcomes, with rationale	Yes		pg 6, line 4-20
**Risk of bias in individual studies**	14	Describe anticipated methods for assessing risk of bias of individual studies, including whether this will be done at the outcome or study level, or both; state how this information will be used in data synthesis	Yes		pg 6, line 23-58, pg 7 line 4-18
DATA					
**Synthesis**	15a	Describe criteria under which study data will be quantitatively synthesized	Yes		pg 7, line 33-54
	15b	If data are appropriate for quantitative synthesis, describe planned summary measures, methods of handling data, and methods of combining data from studies, including any planned exploration of consistency (e.g., I^2, Kendall's tau)	Yes		pg 7, line 33-54
	15c	Describe any proposed additional analyses (e.g., sensitivity or subgroup analyses, meta-regression)	NA		
	15d	If quantitative synthesis is not appropriate, describe the type of summary planned	Yes		pg 7, line 33-54
**Meta-bias(es)**	16	Specify any planned assessment of meta-bias(es) (e.g., publication bias across studies, selective reporting within studies)	Yes		pg 7, line 59-60, pg 8, line 4-7
**Confidence in cumulative evidence**	17	Describe how the strength of the body of evidence will be assessed (e.g., GRADE)		No	
